# Polystyrene-Encapsulated Carbonyl Iron Microcapsules: A Corrosion-Resistant Microwave Absorber

**DOI:** 10.3390/ma18081779

**Published:** 2025-04-13

**Authors:** Ke Gai, Junhe Shi, Wanxun Li, Weisen Liu, Weiping He, Qian Wang, Tong Zhao

**Affiliations:** 1 Key Laboratory of Science and Technology on High-Tech Polymer Materials, Institute of Chemistry, Chinese Academy of Sciences, Beijing 100190, China; 2 Beijing National Laboratory for Molecular Sciences, Beijing 100190, China; 3 Aviation Key Laboratory of Science and Technology on Advanced Surface Engineering, AVIC Manufacturing Technology Institute, Beijing 100024, China; 4 University of Chinese Academy of Sciences, Beijing 100049, China; 5 Structure Corrosion Protection and Control of Aviation Science and Technology Key Laboratory, China Special Vehicle Research Institute, Jingmen 448035, China

**Keywords:** carbonyl iron, microwave absorption, microcapsule, corrosion resistance, coating

## Abstract

Carbonyl iron powder is a widely used microwave-absorbing material due to its numerous advantages. However, carbonyl iron powder is prone to corrosion in high-salt-spray environments, reducing the service life of the composite material and limiting its applications, particularly in marine environments. In this study, we prepared polystyrene-encapsulated carbonyl iron microcapsules via in-situ polymerization and investigated their structure and properties. The results show that the coating of the polystyrene shell did not affect the crystal structure of the carbonyl iron and hardly weakened its electromagnetic properties. Compared to uncoated carbonyl iron powder, polystyrene-encapsulated carbonyl iron microcapsules exhibited superior corrosion resistance in both HCl solution and salt-spray environment. This work offers a potential solution for enhancing the durability of microwave-absorbing material in corrosive environments. With this simple, effective, and low-budget procedure, the cost of microwave-absorbing coating used in marine environments would be significantly reduced.

## 1. Introduction

With the development of modern electronic information technology, there are many great challenges facing mankind, such as electromagnetic pollution prevention and information leakage protection technology. Thus, microwave-absorbing materials (MAMs) have emerged as a promising solution and are widely used [1,2,3,4,5,6]. These materials can significantly reduce electromagnetic pollution while effectively mitigating electromagnetic interference and information leakage [7]. Particularly, equipment covered with MAMs may substantially enhance their electromagnetic wave-absorbing capabilities and avoid detection and tracking by radar systems. Therefore, the exploitation of MAMs is of great significance to the development of contemporary technology.

MAMs, including carbon, graphite [8,9], conductive polymers [10,11], ceramics [12,13], ferrite [14,15], and metal powders [16,17], have been widely investigated. Wherein, carbonyl iron powder (CIP) has been extensively explored owing to its many benefits that include superb wave-absorbing capacity, high microwave permeability, low hysteresis loss, thin matching thickness, good thermal stability, and high saturation magnetization [18,19,20,21,22]. As the application of CIP microwave-absorbing composites in the marine environment continues to expand, poor corrosion resistance has become an urgent problem to be solved [23,24,25]. In the harsh environments of high temperature, high humidity, and salt spray in the atmosphere and in the ocean, CIP microwave-absorbing composites are prone to oxidative corrosion, which seriously affects the performance of the composites and reduces their service life, eventually resulting in significant economic losses. Therefore, many efforts have been made to improve its corrosion resistance property.

To date, the most generally used approach for preserving CIP is coating it with an organic or inorganic shell layer, which is commonly applied via the chemical deposition method [26,27,28,29], sol–gel method [30,31,32,33,34,35,36], and in-situ polymerization process [37,38,39,40,41,42]. The chemical deposition method is used to prepare metal shell layers, such as CIP@Co(Ni) [28]. The CIP with a metal shell generally had strong microwave absorption performance and displayed long-life stability of the microwave absorption performance in seawater. Duan et al. created Fe@AlPO_4_ core–shell composites; due to the low oxygen permeability of the AlPO_4_, the anti-oxidative property of the internal carbonyl iron effectively improved [29].

Due to their thermal stability, CIP microcapsules with SiO_2_ and organosilane shells have been synthesized by the sol–gel method, such as CIP@SiO_2_ [32], CIP@PFOTES [34,35], and CIP@BTSA@OTMS [36]. The favorable hydrophobicity of the SiO_2_ and organosilane layer can hinder the contact of the corrosive medium with CIP to improve the impedance matching of the materials. The in-situ polymerization method was used to microcapsulate CIP with an organic shell layer. For example, Guo et al. synthesized a novel CIP@EP and validated that the epoxy was densely covered on the surface of the CIP [21]. Tang et al. prepared PANI/PVP/CIP core–shell composites, wherein the PVP/PANI overlayer prevented the oxidation of particles and acted as corrosion protection for the CIP [39]. Thus, encapsulation is an effective way to enhance the anti-corrosion property of the CIP.

In this work, CIP was encapsulated by an organic polystyrene layer via an in-situ polymerization method. The properties of the prepared carbonyl iron powder@polystyrene (CIP@PS) microcapsules and the CIP were compared, including the chemical structure, micromorphology, electromagnetic properties, and corrosion resistance ability. The PS shell layer did not affect the crystal structure of the CIP and hardly weakened its electromagnetic properties. Compared to uncoated carbonyl iron powder, CIP@PS microcapsules exhibited superior corrosion resistance in both HCl solution and a salt-spray fog environment. Thus, the microwave absorption coating with CIP@PS can be employed in a saline–fog environment for a longer period.

## 2. Materials and Methods

### 2.1. Materials

Carbonyl iron powder was purchased from Sinopharm Chemical Reagent Co., Ltd. (Shanghai, China). Xylene (AR) and Maleic anhydride (MA, AR) were purchased from Modern Oriental (Beijing) Technology Development Co., Ltd. (Beijing, China). Styrene (AR), Divinylbenzene (50% mixture of isomers), and 2,2′-Azobis (2-methylpropionitrile) (AIBN, 98%) were purchased from Shanghai Macklin Biochemical Technology Co., Ltd. (Shanghai, China).

### 2.2. Preparation of CIP@PS

First, 1.5 g of MA was added to 200 g of xylene at 70 °C and stirred for 0.5 h, and then 50 g of CIP was added and stirred in N_2_ for 1 h. Subsequently, 1 g of styrene, 0.5 g of divinylbenzene, and 0.1 g of AIBN were added and stirred for 7 h at 70 °C to obtain the polymer-shelled CIP-PS.

### 2.3. Characterization

FT-IR spectra of materials were recorded using an FT-IR spectrometer (TENSOR27, Bruker, Karlsruh, Germany) in the range of 4000–400 cm⁻^1^. The X-ray diffraction patterns were obtained by X-ray diffraction (D8 ADVANCE, Bruker, Karlsruh, Germany). The microstructure of materials was observed using SEM (SU8020, Hitachi, Tokyo, Japan), and EDS mappings of the sample were obtained through an Oxford detector attached to the SEM. The Fe^2+^ content in the solution after the corrosion-resistance test was analyzed by ICP-OES (5110, Agilent, Santa Clara, CA, USA). The electromagnetic parameters were measured by a vector network analyzer (E5071C, Agilent, CA, USA). The salt-spray corrosion test was carried out by Q-FOG (SSP1100, H. J. UNKEL, Hongkong, China) with 5% of NaCl solution at 35 ± 1 °C.

## 3. Results and Discussion

### 3.1. Structure and Morphology Analysis

**Figure 1:**
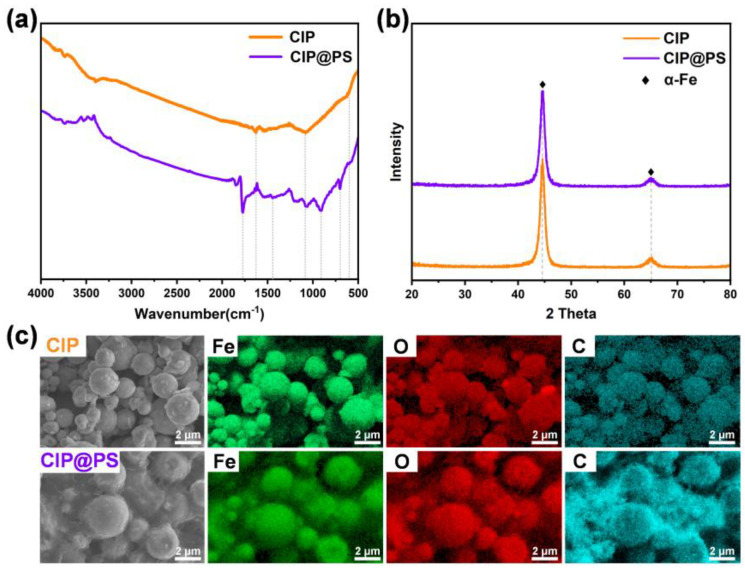
(**a**) FT-IR spectra, (**b**) XRD patterns, and (**c**) SEM and EDS images of CIP and CIP@PS.

Figure 1a shows the FT-IR spectra of CIP and CIP@PS. The stretching vibration of carbonyl iron corresponded to three broad bands located at 1629 cm^−1^, 1084 cm^−1^, and 600 cm^−1^ [19,21,27,40], which were both observed in the FT-IR spectra of CIP and CIP@PS, with a decrease in the amplitude of the signal vibrations brought about by the polymer shell coating. In addition, in the FT-IR spectrum of CIP@PS, a peak at 698 cm^−1^, 908 cm^−1^ and 1450 cm^−1^ corresponded to the C-H and C=C bending vibration from the benzene ring, and the peak at 1772 cm^−1^ was attributed to the C=O bending vibration from maleic anhydride, confirming the presence of a polystyrene shell layer.

As shown in Figure 1b, the X-ray diffraction patterns were carried out to obtain more structural information. Since polystyrene is an organic material with an amorphous structure and does not have characteristic diffraction peaks, the CIP and CIP@PS exhibited the same crystalline peaks. The two crystalline peaks at 44.77° and 65.02° correspond to the (1 1 0) and (2 0 0) crystal planes of α-Fe, respectively; no other phase formed, and the positions of the diffraction peaks did not shift, proving that the crystal structure of the carbonyl iron was not affected by the polystyrene shell. Due to the encapsulation of the polystyrene shell, the diffraction peak of α-Fe of CIP@PS was slightly broader and weaker compared to the CIP.

Figure 1c shows the SEM and EDS mapping images of CIP and CIP@PS. From the SEM images, the diameter of carbonyl iron powder was about 1–3 μm, and the diameter of CIP@PS was slightly increased but still uniform, which proves the successful coating of the polystyrene shell layer. As can be seen from the EDS mapping images, the elements Fe and O in the carbonyl iron powder without the coating shell were uniformly distributed, and the signal of the Fe element appeared to be strong. The distribution of the elements Fe, C, and O in CIP@PS remained uniform, in which the signals of Fe belonging to the carbonyl iron powder were noticeably weakened, while the signal of C was significantly enhanced because of the polystyrene shell layer, demonstrating the successful preparation of the polymer coating.

### 3.2. Wave-Absorption and Corrosion-Resistance Performance

**Figure 2:**
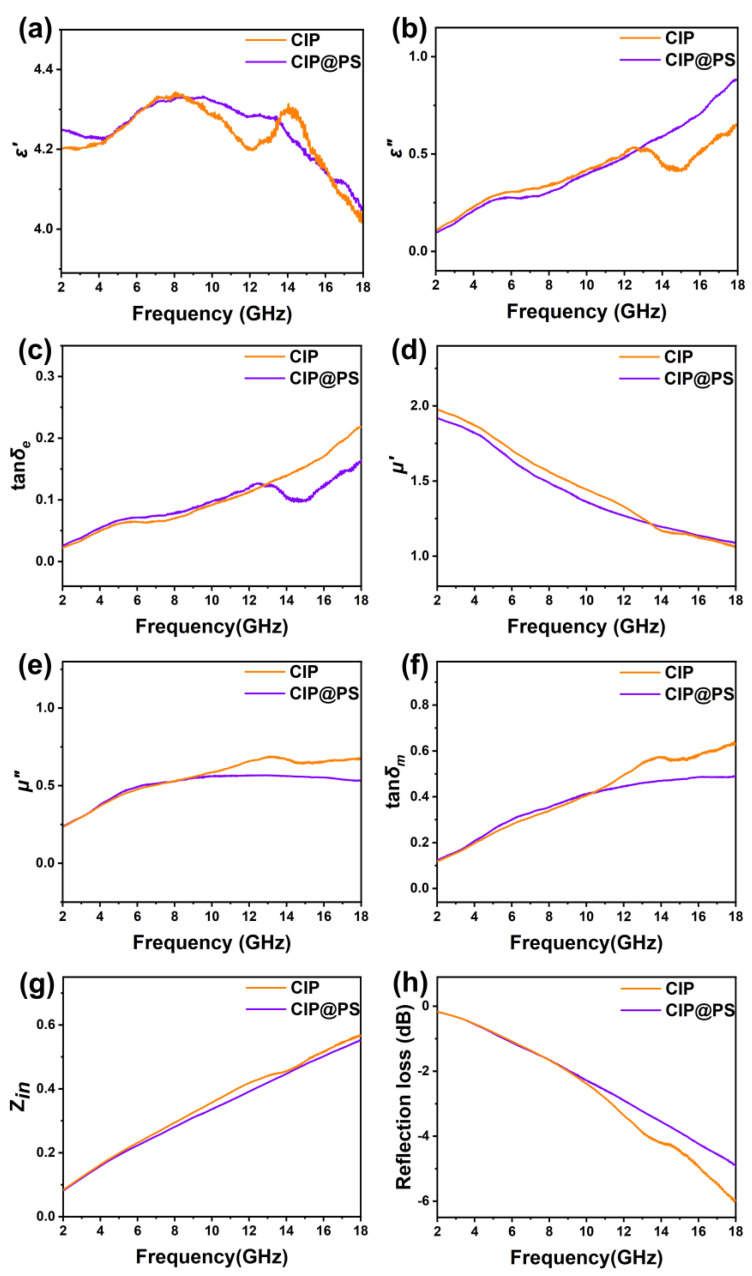
Electromagnetic parameters of CIP and CIP@PS in the frequency range of 2–18 GHz: (**a**) real part of permittivity *ε*′, (**b**) imaginary part of permittivity *ε*″, (**c**) dielectric loss factor tan*δ_e_* (*ε*″/*ε*′), (**d**) real part of permeability *μ*′, (**e**) imaginary part of permeability *μ*″, (**f**) magnetic loss factor tan*δ_m_* (*μ*″/*μ*′), (**g**) input impedance Z_in_, and (**h**) RL curves.

To investigate the microwave absorption performance of CIP and CIP@PS, the electromagnetic parameters were examined with a vector network analyzer. As shown in Figure 2, the real part of the permittivity *ε*′ (Figure 2a) of the carbonyl iron powder was about 4.2, and the dielectric loss angle < 0.15; additionally, the magnetic permeability showed typical dispersion characteristics, with the real part of the magnetic permeability *μ*’ (Figure 2d) decreasing from 1.98 at 2 GHz to 1.06 at 18 GHz, and the imaginary part of the magnetic permeability *μ*″ (Figure 2e) ranging from 0.23 to 0.69, and the magnetic loss angle tan*δ_m_* (Figure 2f) increasing from 0.12 at 2 GHz to 0.63 at 18 GHz, which indicated that the loss capability of the carbonyl iron powder was mainly dependent on the magnetic loss. After coating the polystyrene shell layer, there was no significant change in the dielectric constant of CIP@PS due to the thin coating layer (Figure 2a–c). For the magnetic properties, the change in the magnetic loss angle at 10–18 GHz was most obvious (Figure 2d–f), and the tan*δ_m_* (Figure 2f) of CIP@PS at 10–18 GHz decreased to 0.40–0.49, while that of the carbonyl iron powder was 0.41–0.63, which was mainly attributed to the slight decrease in spatial magnetic density after coating with the polymer.

It is well known that the reflection loss from a coating depends mainly on the electromagnetic loss capability (tan*δ_e_* and tan*δ_m_*) and the impedance matching characteristics of the coating. The closer the normalized impedance value *Z* is to 1, the better the matching characteristics of the coating to the air.

The reflection loss is given by the following relation:

(1) $$Z_{in}=\sqrt{\frac{\mu }{\varepsilon }}\tan h(i\frac{2\pi fd}{c}\sqrt{\mu \varepsilon })$$

(2) $$RL(dB)=20\mathrm{lg}\left|\frac{Z_{in}-1}{Z_{in}+1}\right|$$

where *Z_in_* is the input impedance, *μ* and *ε* are the relative permeability and permittivity of the absorber, respectively, *f* is the frequency of the electromagnetic wave, *d* is the thickness of the absorber, and *c* is the velocity of light in free space.

The calculated *Z_in_* values of both did not change significantly, which indicated that the degradation in the RL performance of CIP@PS within 10–18 GHz mainly originated from the degradation in the magnetic loss performance in this range. The reflection loss performance of the 1.0 mm coating was simulated according to transmission line theory [43,44]. The reflection loss curves for both were in general agreement, and CIP@PS had a weakening of RL values within 1.0 dB compared to CIP within 10–18 GHz.

**Figure 3:**
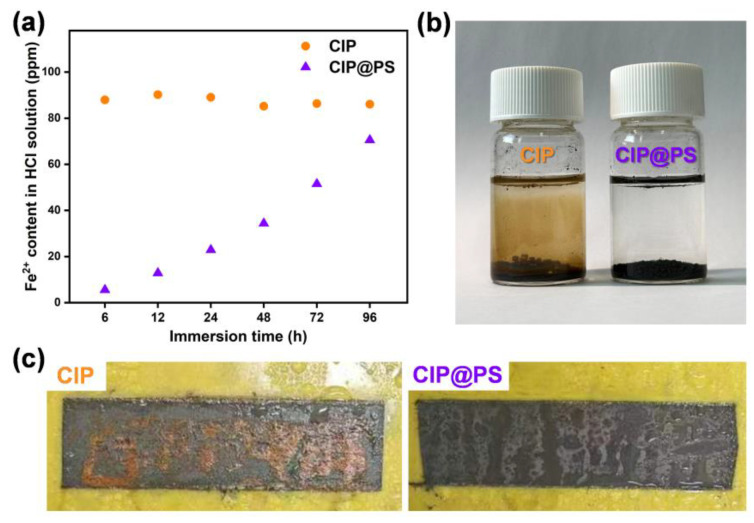
(**a**) The variation curves of Fe^2+^ content with immersion time in HCl solution immersed with CIP and CIP@PS, (**b**) the states of the CIP and the CIP@PS after immersion in HCl solution for 96 h, and (**c**) the experimental results for CIP and CIP@PS after exposure to salt-spray fog for 96 h.

A comparison of the corrosion resistance of CIP and CIP@PS is shown in Figure 3. Firstly, the protection of the polystyrene shell layer on carbonyl iron powder was investigated in an acidic solution. When both were immersed in HCl, the carbonyl iron powder without a coating shell layer was corroded by HCl, resulting in the formation of Fe^2+^ very quickly, while the organic shell layer of CIP@PS hindered the corrosion by HCl. As shown in Figure 3a, the Fe^2+^ content in the HCl solution immersed with CIP reached 80 ppm at 6 h, while the Fe^2+^ content in the HCl solution immersed with CIP@PS was below 10 ppm at 6 h, which was attributed to the protective effect of the dense polystyrene shell layer. Although the Fe^2+^ content in the HCl solution immersed with CIP@PS was increasing slowly with the extension of immersion time, it was still about 70 ppm after 96 h of immersion, which was lower than of carbonyl iron, showing that the polymer shell layer had a protecting effect in HCl. The states of CIP and CIP@PS after 96 h of immersion in HCl are shown in Figure 3b, where the carbonyl iron powder had been thoroughly corroded, presenting a yellow solution and bubbles on the surface. For CIP@PS, on the other hand, no bubbles were observed on the surface and the solution remained clear and transparent. Therefore, this demonstrates that the polystyrene shell layer can effectively improve the corrosion resistance of the material. Then, the corrosion resistance was further investigated by salt-spray corrosion tests of the CIP coating and CIP@PS coating to simulate an actual marine environment. The experimental results are shown in Figure 3c. After 96 h of exposure to the salt-spray environment, the uncoated carbonyl iron revealed apparent rust on its surface, while the CIP@PS, which was coated by an organic shell, remained without rust, indicating that the coating of polystyrene had significantly improved the corrosion resistance of carbonyl iron powder in the salt spray.

## 4. Conclusions

In this work, we fabricated CIP@PS microcapsules by coating a PS shell on the surface of carbonyl iron powder via a simple one-step in-situ polymerization method. The PS shell layer had been proven to be dense enough to enhance the corrosion resistance performance of carbonyl iron powder. Additionally, the PS shell did not significantly weaken the microwave absorption properties. Compared to uncoated carbonyl iron powder, CIP@PS microcapsules exhibited superior long-term stability in both HCl immersion and salt-spray environments for an extended time. This approach can effectively improve the long-term stability of MAMs (especially carbonyl iron powder) under corrosive conditions such as the marine environment. In future work, the surface chemical structure will be systematically optimized to enhance interfacial compatibility with polymeric matrices, thereby expanding the material’s applicability in multifunctional coatings. Furthermore, this cost-effective, facile, and scalable methodology demonstrates significant potential for mitigating corrosion susceptibility in diverse material systems, with promising prospects for deployment in extreme environments.

## Data Availability

The data presented in this study are not publicly available due to privacy and are available on request from the corresponding author upon reasonable request.
